# Gadolinium Enhanced 3D Proton Density Driven Equilibrium MR Imaging in the Evaluation of Cisternal Tumor and Associated Structures: Comparison with Balanced Fast-Field-Echo Sequence

**DOI:** 10.1371/journal.pone.0103215

**Published:** 2014-07-22

**Authors:** Sung Jun Ahn, Mi Ri Yoo, Sang Hyun Suh, Seung-Koo Lee, Kyu Sung Lee, Eun Jin Son, Tae-Sub Chung

**Affiliations:** 1 Department of Radiology, Yonsei University College of Medicine, Seoul, Republic of Korea; 2 Department of Neurosurgery, Yonsei University College of Medicine, Seoul, Republic of Korea; 3 Department of Otorhinolarygology, Yonsei University College of Medicine, Seoul, Republic of Korea; Northwestern University Feinberg School of Medicine, United States of America

## Abstract

**Objectives:**

Although Gadolinium enhanced bFFE is commonly used to evaluate cisternal tumors, banding artifact may interrupt interpretation and adjacent nerve and vessels differentiation is known to be difficult. We analyzed the qualities of Gd enhanced 3D PDDE in the evaluation of cisternal tumors, comparing with bFFE.

**Material and Methods:**

Forty five cisternal tumors (33 schwannoma and 12 meningioma) on both bFFE and PDDE were retrospectively reviewed. For quantitative analysis, contrast ratios of CSF to tumor and tumor to parenchyma (CR_C/T_ and CR_T/P_) on both sequences were compared by paired t-test. For qualitative analysis, the readers gauged the qualities of the two MR sequences with respect to the degree of demarcating cisternal structures (tumor, basilar artery, AICA, trigeminal nerve, facial nerve and vestibulocochlear nerve).

**Results:**

In quantitative analysis, CR_C/T_ and CR_T/P_ on 3D PDDE was significantly lower than that of 3D bFFE (*p*<0.01). In qualitative analysis, basilar artery, AICA, facial nerve and vestibulocochlear nerves were significantly better demarcated on 3D PDDE than on bFFE (*p*<0.01). The degree of demarcation of tumor on 3D PDDE was not significantly different with that on 3D bFFE (*p* = 0.13).

**Conclusion:**

Although the contrast between tumor and the surrounding structures are reduced, Gd enhanced 3D PDDE provides better demarcation of cranial nerves and major vessels adjacent to cisternal tumors than Gd enhanced bFFE

## Introduction

Balanced steady-stated free precession (bSSFP) sequences such as true free induction with steady precession (trueFISP), fast imaging employing steady-state acquisition (FIESTA), and balanced fast field echo (bFFE) are commonly used to evaluate structures in the prepontine cistern and cerebellopontine angle (CPA). This sequence has high spatial resolution and heavily T2 contrast between cerebrospinal fluid (CSF) and other structures, such as nerve, bone and brain parenchyma [1]–[4]. With gadolinium contrast media, it provides excellent visualization of the boundary of the cisternal tumors with surrounding structures because it has inherent T1 contrast [5]–[8].

However, it is difficult to discriminate cranial nerves, small vessels, and skull base structures because all structures except for CSF are outlined as hypo-intense areas [9], [10], while large vessels show hyper-intensities and are confused with surrounding CSF spaces [1], [11]. Furthermore, banding artifact inherent to bSSFP may make it difficult to distinguish structures in the CPA [12], [13]. These are fatal disadvantages of this sequence, because identification of exact relationship between the tumor and its surrounding structures may have implications in preventing unnecessary hemorrhage during surgery as well as for neural preservation.

3D proton density driven equilibrium (3D PDDE) may be used for vessel wall imaging because it provides excellent blood suppression and MR cisternographic features [14]. DRIVE pulses at the echo train of 3D proton density push residual transverse magnetization back to the longitudinal axis, providing T2 contrast with a higher signal from CSF [15], [16]. We incidentally found that cisternal tumors show strong enhancement with clear margin and associated structures are discernible with consistent signal intensities on Gd 3D PDDE.

The aim of our study is to analyze the qualities of Gd enhanced 3D PDDE in the evaluation of cisternal tumors and associated structures, comparing with Gd enhanced bFFE.

## Materials and Methods

### Patients

The protocol for this retrospective study was approved by Gangnam Severance Hospital, institutional review board and informed consent for this retrospective study was not required. Patient records and information were anonymized and de-identified prior to analysis. We identified 45 patients (17 men and 28 women; age range 42–78 years, mean age 56.8 years) who have schwannoma (n = 33) or meningioma (n = 12) from our medical record system between May 2013 and Jan 2014. Inclusion criteria was as follows; (1) Gd enhanced MRI sequences, which they performed, should include both bFFE and 3D PDDE after Gd injection. (2) Tumor location was prepontine cistern(n = 13) or CPA (n = 32). The diagnosis was based on morphological findings of MRI as follows; If the mass showed extension along the course of cranial nerves with or without internal cysts and hemorrhage, it was diagnosed as the schwannoma. If the mass showed broad base with dural ‘tail’, it was diagnosed as the meningioma [17], [18]. There were no equivocal cases with diagnosis under morphological findings. The mean size of cisternal tumors was 20.2 mm (range, 5.9∼43.5 mm).

### Imaging acquisition

Gd enhanced MRI was performed using 3T MR units (Achieva; Philips Medical Systems, Best, Netherlands) and a 32-channel sensitivity encoding (SENSE) head coil on all patients. T2 axial turbo spin echo images (TR/TE = 6090/100 ms, thickness = 2 mm, gap = 0.2 mm, field of view = 230×230 mm, matrix = 256×223), T2 coronal turbo spin echo images (TR/TE = 3000/100 ms, thickness = 2 mm, gap = 0.1 mm, field of view = 200×200 mm, matrix = 512×256) were acquired. After injecting 0.1 mmol/kg gadobutrol, 3D bFFE (TR/TE = 6.7/2.7 ms, flip angle = 45, thickness = 0.4 mm, field of view = 180×180 mm, matrix = 448×450 [reconstructed into 480×480], number of signal averaged = 5, acquisition time = 7–8 min) and 3D PDDE (TR/TE = 2000/32.2 ms, thickness = 0.4 mm, field of view = 180×180 mm, matrix  = 480×480, number of signal averaged = 1, echo train length = 63, acquisition time = 8∼9 min) were obtained. A variable-flip-angle refocusing plus train was used with α min of 50 and α max of 120. In both sequences, the axial plane was scanned parallel to the orbitomeatal line. Oblique sagittal and coronal images were reconstructed.

### Quantitative analysis

A radiology resident (M.R.Y) drew three different circular ROIs (area = 10 mm^2^) within the tumor, avoiding necrosis and hemorrhage. The average value of three different ROIs was regarded as the signal intensity of tumor (SI_T_). The signal intensity of CSF (SI_C_) was measured with the same method which draw ROIs in the ipsilateral cistern, avoiding adjacent vessel and nerves. The signal intensity of parenchyma (SI_P_) was measured with the same method, drawing ROIs in the pons. Contrast ratio of CSF to tumor (CR_C/T_) was defined as the signal intensity of CSF over that of tumor. Contrast ratio of tumor to parenchyma (CR_T/P_) was defined as the signal intensity of tumor over that of pons. Another reader, a board certified neuroradiologist (S.J.A), independently measured SI_T_, SI_C_, SI_P_ ,CR_C/T_ and CR_T/P_, Average values between the two readers were used for further analysis. We compared SI_T_, SI_C_, SI_P_ ,CR_C/T_ and CR_T/P_ between the two sequences.

### Qualitative analysis

Two readers (S.H.S, T.S.C) independently evaluated 45 cisternal tumors and associated structures for both 3D bFFE and 3D PDDE. The two readers were board-certified radiologists with 7 and 21 years of reading brain MRIs respectively. There were two sessions with 2-week intervals. At the first session, the first reader was asked to review 3D bFFE and the second reader was asked to review 3D PDDE. At the second session, reviewers evaluated the other sequences to reduce bias. The reviewers gauged the quality of two MR sequences with respect to the degree of demarcating cisternal structures. The evaluated structures were as follows: 1) tumor. 2) basilar artery. 3) ipsilateral anterior inferior cerebellar artery (AICA). 4) ipsilateral facial nerve. 5) ipsilateral vestibulocochlear nerve. 6) ipsilateral trigeminal nerve. Reviewers used a three-point scale system for evaluation: Grade 1 = The evaluated structure was “not” discriminated from surrounding structures in any plane. Grade 2 = The evaluated structure was discriminated from surrounding structures but contrast is not “good” Grade 3 = The evaluated structures were clearly discriminated from surrounding structures and have good contrast. In addition, the resident was requested to record existence of MR banding artifacts. If banding artifacts extended into prepontine and CPA cistern and influenced interpretation, they were also recorded.

### Statistical analysis

Statistical analyses were performed using SPSS version 20.0 (SPSS Inc., Chicago, IL, USA).

For quantitative analysis, The inter-observer agreement between the two readers was evaluated by using the intraclass correlation coefficient(ICC) [19] and the ICC greater than 0.75 was considered to represent good agreement [20]. SI_T_, SI_C_, SI_P_, CR_C/T_ and CR_T/P_ from the both sequences were compared by paired t-test. For qualitative analysis, inter-observer agreement was analyzed by kappa statistics. Visual grades by reviewer 1 were regarded as representative values because of excellent inter-observer agreement. Comparison of visual grades between two sequences were assessed by McNemar's test. P<0.05 was considered statistically significant.

## Results

### Quantitative analysis

**Figure 1:**
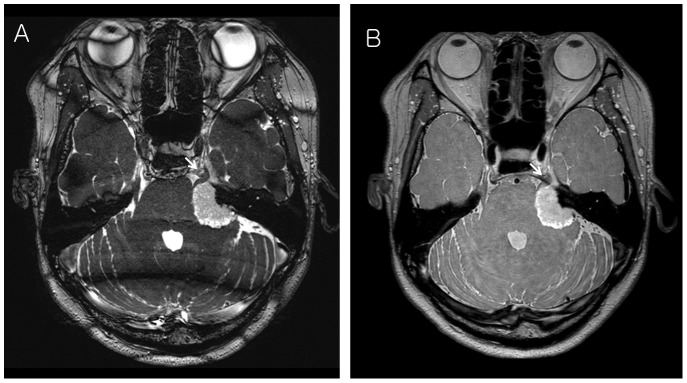
A 42-year-old female with left petrous apex meningioma. (A) The CR_C/T_ was 2.04 and CR_T/P_ was 2.85 on Gd enhanced 3D bFFE. Tumor is well differentiated from brain parenchyma, CSF space and petrous bone (visual scores of two readers : 3). The left trigeminal nerve is well delineated (white arrow). (B) The CR_C/T_ was 1.06 and CR_T/P_ was 1.44 on Gd enhanced 3D PDDE. Tumor is well differentiated from brain parenchyma, CSF space and petrous bone (visual scores of two readers : 3). The left trigeminal nerve is also well delineated (white arrow).

SI_T_, SI_C_, SI_P_, CR_C/T_ and CR_T/P_ in both sequences are summarized in Table 1. SI_T_ in Gd 3D PDDE was significantly higher than SI_T_ in Gd enhanced 3D bFFE(1445.36±242.82 for 3D PDDE; 962.35±179.25 for 3D bFFE, p<0.01). SI_C_ in Gd 3D PDDE was significantly lower than SI_C_ in Gd 3D bFFE(1576.26±139.27 for 3D PDDE; 1945±72.4 for 3D bFFE, p<0.01). SI_P_ in Gd 3D PDDE was significantly higher than SI_P_ in Gd 3D bFFE(1021.87±84.07 for 3D PDDE; 315.53±27.72 for 3D bFFE, p<0.01). CR_C/T_ in Gd 3D PDDE is significantly lower than CR_C/T_ in Gd enhanced 3D bFFE (1.12±0.24 for 3D PDDE; 2.08±0.33 for 3D bFFE, P<0.01). CR_T/P_ in Gd enhanced 3D PDDE is significantly lower than CR_T/P_ in Gd enhanced 3D bFFE (1.42±0.21 for 3D PDDE ; 3.07±0.65 for 3D FFE, P<0.01) (Fig. 1). The inter-observer agreements in SI_T_, SI_C_, SI_P_ ,CR_C/T_ and CR_T/P_ were excellent (ICCs >0.78)

**Table 1:** Comparison of signal intensity and contrast ratios of tumor, CSF and parenchyma between Gd enhanced bFFE and 3D PDDE.

	bFFE	ICC	3D PDDE	ICC	*p*
SI_T_	962.35±179.25	0.91	1445.36±242.82	0.94	<0.01
SI_C_	1945.37±72.4	0.78	1576.26±139.27	0.95	<0.01
SI_P_	315.53±27.72	0.85	1021.87±84.07	0.88	<0.01
CR_C/T_	2.08±0.33	0.95	1.12±0.24	0.93	<0.01
CR_T/P_	3.07±0.65	0.92	1.42±0.21	0.95	<0.01

Note - SI_T_ indicates the signal intensity of tumor, SI_C_ indicates the signal intensity of CSF, SI_P_ indicates the signal intensity of parenchyma, CR_C/T_ indicates the ratio of SI_C_ to SI_T_, CR_T/P_ indicates the ratio of SI_T_ to SI_P._ ICC indicates intraclass correlation coefficient.

### Qualitative analysis

**Figure 2:**
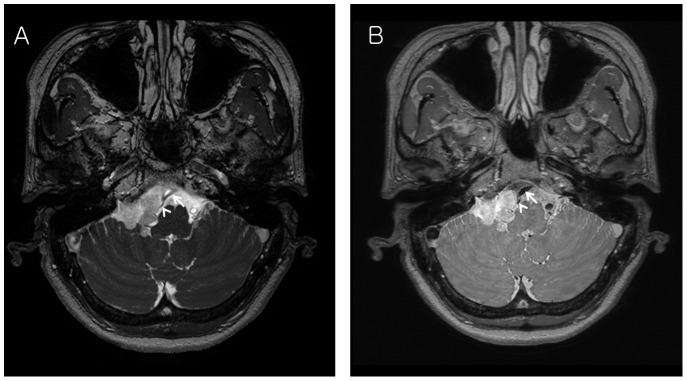
A 54-year-old female with a schwannoma in the right CPA. (A) On Gd enhanced 3D bFFE axial image, basilar artery (white arrow) and right AICA (arrow head) adjacent to tumor border are not demarcated due to various signals from vessels. (B) On Gd enhanced 3D PDDE, basilar artery (white arrow) and right AICA (arrow head) adjacent to tumor border are clearly visualized due to excellent black blood imaging.

Visual grading of demarcation of cisternal anatomical structures in Gd enhanced 3D bFFE and 3D PDDE is summarized in Table 2. The cisternal tumors were well discriminated from surrounding structures in both sequences and the visual grading scores were not significantly different between both sequences (p = 0.13). Ipsilateral trigeminal nerve(CN V) was well demarcated in both sequences without significant difference. However, in discrimination of basilar artery and ipsilateral AICA from surrounding structures, 3D PDDE was significantly better than 3D bFFE. 3D PDDE had more grade 3 scores , while having less grade 1 and 2 scores, compared with 3D bFFE (grade 3 for basilar artery: 45/45 (100%) for 3D PDDE *vs* 7/45 (15.6%) for 3D bFFE, *p*<0.01; grade 3 for AICA: 26/45 (57.8%) for 3D PDDE *vs* 7/45 (15.6%), *p*<0.01, Fig. 2). In discrimination of facial nerve and vestibulocochlear nerve from surrounding structures, 3D PDDE was significantly better than 3D bFFE. 3D PDDE had more grade 3 scores , while having less grade 1 and 2 scores, compared with 3D bFFE (grade 3 for facial nerve: 27/45 (60%) for 3D PDDE *vs* 15/45 (33.3%) for 3D bFFE, *p*<0.01; grade 3 for vestibulocochlear nerve: 30/45 (66.7%) for 3D PDDE *vs* 15/45(33.3%), *p*<0.01).

**Table 2:** Visual grading of demarcation of cisternal anatomical structures in Gd enhanced 3D bFFE and 3D PDDE.

		bFFE	kappa		3D PDDE	kappa	*p*
CPA tumor	Grade 1	0	0.98	Grade 1	0	0.92	0.13
	Grade 2	14(31.1%)		Grade 2	22(48.9%)		
	Grade 3	31(68.9%)		Grade 3	23(51.1%)		
Basilar artery	Grade 1	15(33.3%)	0.84	Grade 1	0	1.00	<0.01
	Grade 2	23(51.1%)		Grade 2	0		
	Grade 3	7(15.6%)		Grade 3	45(100%)		
AICA	Grade 1	23(51.1%)	0.71	Grade 1	15(33.3%)	0.98	<0.01
	Grade 2	15(33.3%)		Grade 2	4(8.9%)		
	Grade 3	7(15.6%)		Grade 3	26(57.8%)		
CN V	Grade 1	0	1.00	Grade 1	0	1.00	
	Grade 2	0		Grade 2	0		
	Grade 3	45(100%)		Grade 3	45(100%)		
CN VII	Grade 1	22(48.9%)	0.92	Grade 1	15(33.3%)	0.87	<0.01
	Grade 2	8(17.8%)		Grade 2	3(6.7%)		
	Grade 3	15(33.3%)		Grade 3	27(60%)		
CN VIII	Grade 1	22(48.9%)	0.92	Grade 1	8(17.8%)	0.92	<0.01
	Grade 2	8(17.8%)		Grade 2	7(15.6%)		
	Grade 3	15(33.3%)		Grade 3	30(66.7%)		

Note- Grade 1 = The evaluated structure was “not” discriminated from surrounding structures in any axial plane. Grade 2 = The evaluated structure was discriminated from surrounding structures but contrast is not “good”. Grade 3 = The evaluated structures were clearly discriminated from surrounding structures with good contrast. kappa indicates the interobserver agreement between two readers. AICA indicates anterior inferior cerebellar artery. CN indicates cranial nerve.

The interobserver agreements between two readers were either good or excellent in grading the two sequences (kappa>0.71).

**Figure 3:**
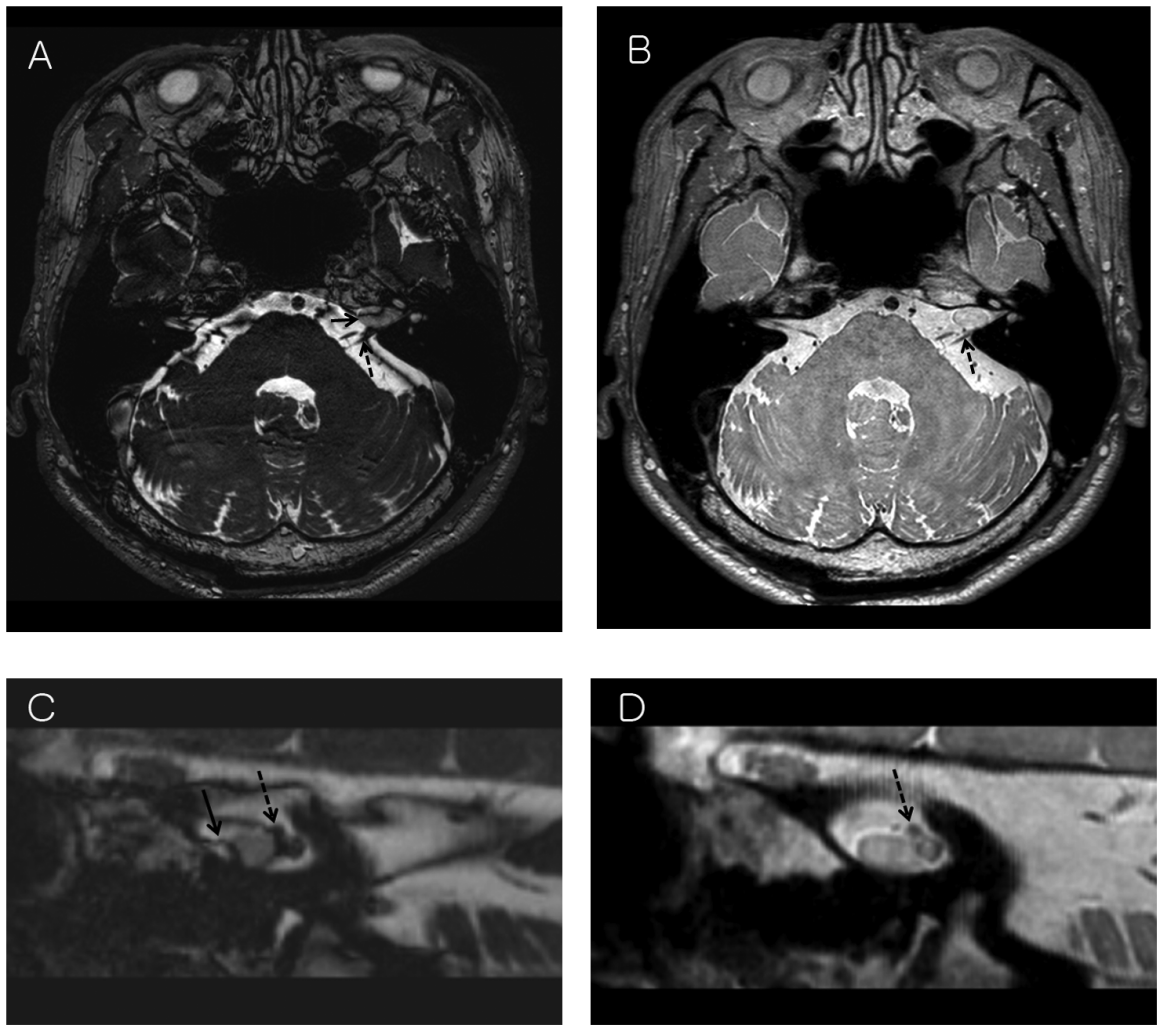
A 61-year-old female with a schwannoma in the left internal auditory canal. (A) On Gd enhanced 3D bFFE, the anterior margin of tumor is not well demarcated due to banding artifact (arrow). Facial and vestibulocochlear nerves are not clearly visualized due to banding artifact (dotted arrow). (B) On Gd enhanced 3D PDDE, the boundary of tumor is clear. Facial and vestibulocochlear nerves are well visualized without banding artifact (dotted arrow). (C) Tumor and cranial nerves are not clearly demarcated on 3D bFFE reconstruction image perpendicular to the left internal auditory canal. (D) They are clearly demarcated on 3D PDDE reconstruction image.

Twenty five out of 45 lesions (56%) showed banding artifacts on 3D bFFE. Seventeen of 45 lesions (38%) had severe banding artifacts that could interrupt interpretation(Fig. 3). While, there was no banding artifact on 3D PDDE

## Discussion

Although contrast between tumor and surrounding structures (CSF and brain parenchyma) on Gd enhanced 3D PDDE are significantly lower than Gd enhanced 3D bFFE, qualitative gauge of cisternal tumor on Gd enhanced 3D PDDE were not significantly different with that on Gd enhanced bFFE. The degree of tumor demarcation is affected by contrast with CSF and brain parenchyma as well as adjacent vessels and nerves. Contrary to bFFE, on Gd enhanced 3D PDDE, adjacent nerves and vessels were clearly demarcated which has a clinical impact in determining surgical plan. Moreover, the excellent MR cisternographic features without banding artifact may compensate the relatively low contrast ratios on Gd enhanced 3D PDDE.

On Gd enhanced 3D PDDE, basilar artery and AICA adjacent to cisternal tumors were clearly demarcated. 3D PDDE provides robust flow independent black blood imaging showing homogenous dark vessel signal intensity [21], [22]. On the contrary, large vessels on 3D bFFE show hyper signal intensities which may cause confusion with surrounding bright CSF. The signal intensity difference between nerve and vessels on Gd enhanced 3D PDDE makes it easier differentiating nerve from vessels. Lower cranial nerves are confused with adjacent small vessels on bFFE, because both are demonstrated as hypo-intensity. However, cranial nerves showed relatively higher signal than vessels on 3D PDDE because the signal intensity depends on proton density.

Another major drawback of bFFE is the banding artifact which is a linear band of low signal inherent to 3D bFFE [23], [24]. Seventeen of 45 lesions (38%) had severe banding artifacts extending into cistern that mimicked cranial nerves and vessels even though relatively short TR and proper shimming were performed. However, 3D PDDE did not show any banding artifact because this technique is in the spin echo family and is less sensitive to field inhomogeneity.

This study has some limitations. Firstly, for quantitative analysis of contrast between tumor and surrounding structures, we calculated CR instead of contrast-to-noise ratio (CNR). This is because a direct measurement of noise was impossible with a SENSE technique that might induce artificial suppression of background noise [25]. Secondly, the cohort of this study is limited to patients with schwannoma and meningioma. The usefulness of Gd enhanced 3D PDDE is questionable in the evaluation of other cisternal lesions such as epidermoid cyst, ependymoma and cavernous malformations. Thirdly, we used single dose of Gd contrast. However, optimal dose of Gd to maximize the contrast between tumor and surrounding structure on 3D PDDE was not determined. Further study is necessary for the optimal dose of Gd.

In conclusion, although the contrast between tumor and surrounding structures are reduced, Gd enhanced 3D PDDE provides better demarcation of cranial nerves and major vessels adjacent to cisternal tumors than Gd enhanced bFFE.
